# Analysis of spot urine biomarkers and association with body weight in Japanese elementary schoolchildren

**DOI:** 10.1007/s00431-022-04604-7

**Published:** 2022-09-13

**Authors:** Nozomi Takemoto, Jasmine Millman, Tsugumi Uema, Fusae Yamakawa, Shiki Okamoto, Mari Mori, Hideki Mori, Koshi Nakamura, Yukio Yamori, Hiroaki Masuzaki

**Affiliations:** 1 Division of Endocrinology, Diabetes and Metabolism, Hematology, Rheumatology, Second Department of Internal Medicine), Graduate School of Medicine, University of the Ryukyus, 207 Uehara, Nishihara, Okinawa 903-0215 Japan; 2 Department of Health Management, School of Health Studies, Tokai University, Kanagawa, Japan; 3 Institute for World Health Development, Mukogawa Women’s University, Hyogo, Japan; 4 Department of Public Health and Hygiene, Graduate School of Medicine, University of the Ryukyus, Okinawa, Japan

**Keywords:** Spot urine, Salt, Magnesium, Body mass index, Obesity

## Abstract

Childhood obesity is rapidly increasing worldwide and is largely the consequence of adoption of unhealthy diets excessive in calories and salt (NaCl) as well as devoid in pivotal micronutrients such as potassium (K) and magnesium (Mg). Education-based programs aiming to encourage healthy food knowledge and behaviors are crucial at a young age, and for this purpose, convenient ways to assess daily dietary intake are warranted. We therefore attempted to evaluate the dietary intake of Okinawan schoolchildren in Japan by analyzing a series of biomarkers in morning spot urine samples and explore whether these biomarkers correlate with body weight and a series of metabolic parameters. We enrolled 98 third-grade elementary schoolchildren in Okinawa, Japan. Morning spot urine samples were collected and analyzed using high-performance liquid chromatography (HPLC) to assess dietary intake. We found that estimated daily NaCl intake was higher in obese/overweight children as compared to healthy-weight children (*p* = 0.0001). There was also a significant positive correlation between body mass index (BMI) and NaCl intake (Spearman) (*ρ* = 0.45, *p* < 0.0001) and a negative correlation between BMI and Mg/Cr (*ρ* =  −0.27, *p* = 0.01). Furthermore, Na/K ratio was higher in samples collected on Monday (weekend) as compared to samples collected on Thursday or Friday (weekday) (*p* < 0.0001).

*Conclusion*: Via the use of morning spot urine analyses, our results show that NaCl intake was associated with obesity, and Mg excretion negatively correlated with BMI in Japanese schoolchildren, highlighting the potential role of these micronutrients in maintaining a healthy body weight.

**Table:** 

**What is Known:** *•Overweight and obesity are largely due to excessive consumption of calories and positively correlated with salt (NaCl) intake.* *•Spot urine methods are convenient for assessing the nutritional needs and targeting prevention programs in children.*	
**What is New:** *•Utilizing morning spot urine analyses, estimated NaCl intake is positively correlated and Mg/Cr negatively correlated with BMI in Okinawan schoolchildren.* *•As estimated via morning spot urine samples, a greater proportion of children likely exceeds the recommended NaCl intake on the weekend as compared to weekday.*

**What is Known:**

*•Overweight and obesity are largely due to excessive consumption of calories and positively correlated with salt (NaCl) intake.*

*•Spot urine methods are convenient for assessing the nutritional needs and targeting prevention programs in children.*

**What is New:**

*•Utilizing morning spot urine analyses, estimated NaCl intake is positively correlated and Mg/Cr negatively correlated with BMI in Okinawan schoolchildren.*

*•As estimated via morning spot urine samples, a greater proportion of children likely exceeds the recommended NaCl intake on the weekend as compared to weekday.*

## Introduction

The prevalence of childhood obesity has been swiftly increasing worldwide. Recent global estimates suggest that approximately 340 million children and adolescents aged 5–19 years are classified as overweight or obese [1], with several studies reporting that approximately 40–50% of obese children go onto develop obesity later in adulthood [2–4], putting this group at an elevated risk for developing type 2 diabetes and related cardiometabolic diseases later in life [5].

Although Japan has experienced a relatively low prevalence of obesity compared to other developed countries, Okinawa, a prefecture in the southernmost part of Japan, once renowned as being home to some of the healthiest and longest living people in the world, is now facing a stark increase in obesity and metabolic diseases in both adults and children [6]. This undesirable trend has resulted in an expedition decline in life expectancy in Okinawan people [7]. According to the School Health Survey Japan, approximately 9.2% of 8-year-old boys and girls in Okinawa are obese [8], which is considerably higher compared to the national average of 8.2% (boy) and 6.9% (girl) [9]. Reasons for such an alarming figure largely stem from westernization of the food culture, with increased availability and consumption of energy-dense, high salt– (sodium chloride, NaCl) containing foods such as fried foods and pastries and displacement of the traditional Okinawan diet naturally abundant in micronutrient-rich plant foods such as seaweeds, sweet potato, soy products, and moderate amounts of fish and seafood [7].

Excessive intake of NaCl is one of the main dietary factors diving increases in high blood pressure and cardiovascular diseases [10], with its intake found to be positively correlated with BMI independent of energy intake in adults [11]. Currently, Japanese people typically consume ~10 g per day of NaCl, more than twice as much as the upper 5 g daily limit advised by the World Health Organization [12, 13]. Along with reducing NaCl, increasing intake of micronutrients such as potassium and magnesium can also positively impact blood pressure and associated disease risks [14]. Given that obesity and related diseases often start to develop in early childhood, methods that accurately capture dietary intake profile and are easy to implement are warranted for assessing the nutritional needs and appropriately targeting prevention programs.

Although accurate execution of dietary records and food frequency questionnaire methods require both skilled professionals and participant’s cooperation, assessment of biomarkers in urine offers a convenient and reliable alternative to assess dietary intake of specific nutrients, especially as a public health strategy [12]. In this context, we aimed to assess urinary biomarkers associated with nutrient intake utilizing morning spot urine samples of Okinawan elementary schoolchildren and explore the potential links with obesity.

## Methods

### Study design and participants

The present study was carried out in a cross-sectional fashion, recruiting in 231 third-grade elementary schoolchildren, 8–9 years old, from two elementary schools in Okinawa prefecture, Japan, from November 2019 to January 2020. A total of 98 children (49 boys and 49 girls) were enrolled and asked to provide spot urine samples at four time points as well as measuring anthropometric indices and blood pressure. Exclusion criteria were as follows: (1) presence of acute or chronic kidney disease and or (2) bronchial asthma and the relevant regular use of corticosteroids for the treatment. None of the children met the exclusion criteria. However, we excluded 17 children (5 boys and 12 girls) who provided only one sample across the four time points. Consequently, the remaining 81 children (44 boys and 37 girls) were considered eligible study participants and included in the subsequent analyses.

Ethics approval was obtained by the Ryukyu University Human Research Ethics Committee (approval number: 360, 25 October 2019). Written informed consent was obtained from all participants and their parent (or legal guardians). This study was carried out in accordance with the Helsinki Declaration.

### Assessment of a series of biomarkers in urine

Spot urine samples were collected on Monday mornings to assess weekend eating habits, and Thursday or Friday mornings to assess weekday eating habits. Urine samples were collected over a total of four time points: 1st, Monday November 2019; 2nd, Thursday or Friday, November 2019; 3rd, Monday January and 2020; and 4th, Thursday or Friday, November 2020.

Each spot urine sample was collected in 10-mL tubes at participants’ home, brought to each school and stored at a 4 °C. Samples were then transferred into either an 8-mL tubes or 1-mL tube. Eight-mL samples were stored at 4 °C and 1-mL samples were stored at −80 °C. Eight-mL tube samples were sent to FALCO Biosystems, Ltd. (Tokyo, Japan) to determine the level of pH, urea nitrogen (UN), creatinine (Cr), sodium (Na), potassium (K), and magnesium (Mg). One-mL tube samples were sent to Mukogawa Women’s University (Hyogo, Japan) for analyses of isoflavones (Iso) and taurine (Tau) by HPLC. Briefly, HPLC analyses were performed to assess the urinary value of the common dietary isoflavonoids including daidzein, genistein, and glycitein as previously described, and the sum of daidzein, genistein, glycitein, and equol was calculated as the total concentration of Iso [15, 16]. Salt intake (NaCl [g/day]) was estimated using Tanaka et al.’s equation [17].

### Measurements of anthropometric indices and blood pressure

Body height and weight were measured, and body mass index (BMI) was computed as weight (kg) per the square of height (m). BMI values were converted to age-adjusted and sex-adjusted BMI z-scores from WHO References 2007 [18]. According to the WHO BMI-for-age growth charts, children were classified as underweight (less than −2SD), normal weight (−2SD to less than 1SD), overweight (1SD, equivalent to BMI 25 kg/m^2^ at 19 years, to less than 2SD) or obese (over 2SD, equivalent to BMI 30 kg/m^2^ at 19 years). Blood pressure (BP) was measured in the seated position using an automatic manometer (HBP-1300, Omron Healthcare Co., Ltd, Kyoto, Japan) on three separate occasions over the course of the study by trained researchers.

### Statistical analysis

For participants providing multiple urine samples, we calculated the average of concentration of specific biomarkers from 1st to 3rd urine samples on Monday to assess weekend dietary intake (A), and the average of concentration of specific biomarkers from 2nd to 4th urine samples on either Thursday or Friday to assess weekday dietary intake (B). For participants providing a single urine sample on either 1st or 3rd time point, as well as on either 2nd or 4th time point, the concentration of specific urinary biomarkers from the single urine sample was used for (A) and (B), respectively. Taken together, we used the average of (A) and (B) as the result of specific urinary biomarkers and NaCl.

Data regarding subject characteristics are presented collectively as well as separately for boys and girls. Urinary biomarkers were also compared between healthy weight and obese/overweight children and are shown. Data are expressed as mean ± standard deviation or median and 25th and 75th percentiles. Unpaired *t*-test was used for normal distribution data, while Wilcoxon test was used for skewed distribution data. Spearman’s rank correlation coefficients were used to assess correlations between each urinary biomarker and BMI. A similar correlation method was performed as a sensitivity analysis utilizing participants’ urine samples that were collected across all of four time points. Finally, biomarkers in urine samples were compared between weekend and weekday to examine whether variation of eating habits may be potentially influenced by school lunch or “Kyushoku.”

All probability values were two-tailed, and the significance level was set at *p* < 0.05. All statistical analyses were carried out using standard software packages (JMP pro version 15; SAS Institute Inc., Cary, NC, USA).

## Results

### Characteristics of study participants

The average weight of the children was 29.4 ± 5.5 kg, and the average height was 131.6 ± 5.8 cm, respectively. No statistical differences in parameters were observed between boys and girls (Table 1). According to BMI z-score, 4 boys were classed as obese, and 6 boys and 3 girls were classed as overweight. There were no participants classed as underweight. However, some urinary biomarkers reflecting dietary intake showed statistically significant differences between boys and girls, including Na/Cr (boys 124.2 mEq/gCr versus girls 109.0 mEq/gCr, *p* = 0.01) and K/Cr (boys 28.5 mEq/gCr versus girls 23.8 mEq/gCr, *p* = 0.0085) (Table 2).

**Table 1:** Characteristics of the study participants

	Total (*n* = 81)	Boys (*n* = 44)	Girls (*n* = 37)	*p* value
Height (cm)	131.6 ± 5.8	131.6 ± 6.1	131.7 ± 5.5	*0.93*
Weight (kg)	29.4 ± 5.5	30.1 ± 6.4	28.6 ± 4.0	*0.21*
BMI (kg/m^2^)	16.9 ± 2.2	17.2 ± 2.6	16.4 ± 1.6	*0.10*
BMI z-score^a^	0.16 ± 1.0	0.3 ± 1.1	-0.05 ± 0.8	*0.09*
Obese (*n*)^b^	4	4	0	
Overweight (*n*)^c^	9	6	3	
Underweight (*n*)^d^	0	0	0	
Systolic BP (mmHg)	104 ± 9	105 ± 9	103 ± 9	*0.32*
Diastolic BP (mmHg)	57 ± 6	57 ± 7	57 ± 6	*0.70*

Values are shown as mean ± standard deviation (SD). Unpaired *t*-test was used to compare each characteristic in each sex group

^a^BMI values were converted to age-adjusted and sex-adjusted BMI z-score according to WHO 2007 references [18]

^b^Obese: > +2SD (equivalent to BMI 30 kg/m^2^ at 19 years)

^c^Overweight: > +1SD (equivalent to BMI 25 kg/m^2^ at 19 years)

^d^Underweight: < −2sd

**Table 2:** Urine biomarkers and estimated salt (NaCl) intake in boys and girls

	Total (*n* = 81)	Boys (*n* = 44)	Girls (*n* = 37)	*p* value
pH	6.0 (5.875–6.25)	6.0 (5.875–6.26)	6.0 (5.8125–6.4375)	*0.68*
UN (mg/dL)	1234.8 ± 271.0	1269.8 ± 284.9	1193.2 ± 251.0	*0.21*
Cr (mg/dL)	120.7 ± 37.8	114.3 ± 36.6	128.3 ± 6.3	*0.10*
Na/Cr (mEq/gCr)	118.2 (93.0–156.3)	124.2 (98.4–174.4)	109.0 (78.8–131.1)	*0.01*
K/Cr (mEq/gCr)	25.5 (21.5–38.5)	28.5 (22.9–42.1)	23.8 (20.6–29.6)	<*0.01*
Mg/Cr (mEq/gCr)	13.7 (11.7–16.2)	13.7 (11.3–17.0)	13.4 (11.9–15.7)	*0.99*
NaCl (g/day)	5.0 (4.3–5.9)	5.1 (4.3–6.4)	4.6 (4.3–5.6)	*0.07*
Na/K	4.6 (3.7–6.4)	4.6 (3.7–6.2)	4.6 (3.6–6.4)	*0.95*
	Total (*n* = 75)	Boys (*n* = 42)	Girls (*n* = 33)	*p* value
Iso/Cr (nmol/mgCr)	19.1 (10.3–34.6)	18.2 (10.1–37.4)	19.8 (11.2–32.7)	*0.75*
Tau/Cr (nmol/mgCr)	762.5 (550.0–1040.4)	737.8 (497.1–985.3)	766.7 (569.4–1065.1)	*0.41*

Values are presented as median (25th and 75th percentiles) or mean ± standard deviation (SD). Wilcoxon test or unpaired *t*-test was used to compare each characteristic in each sex group

### Anthropometric indices and urinary biomarkers

**Fig. 1:**
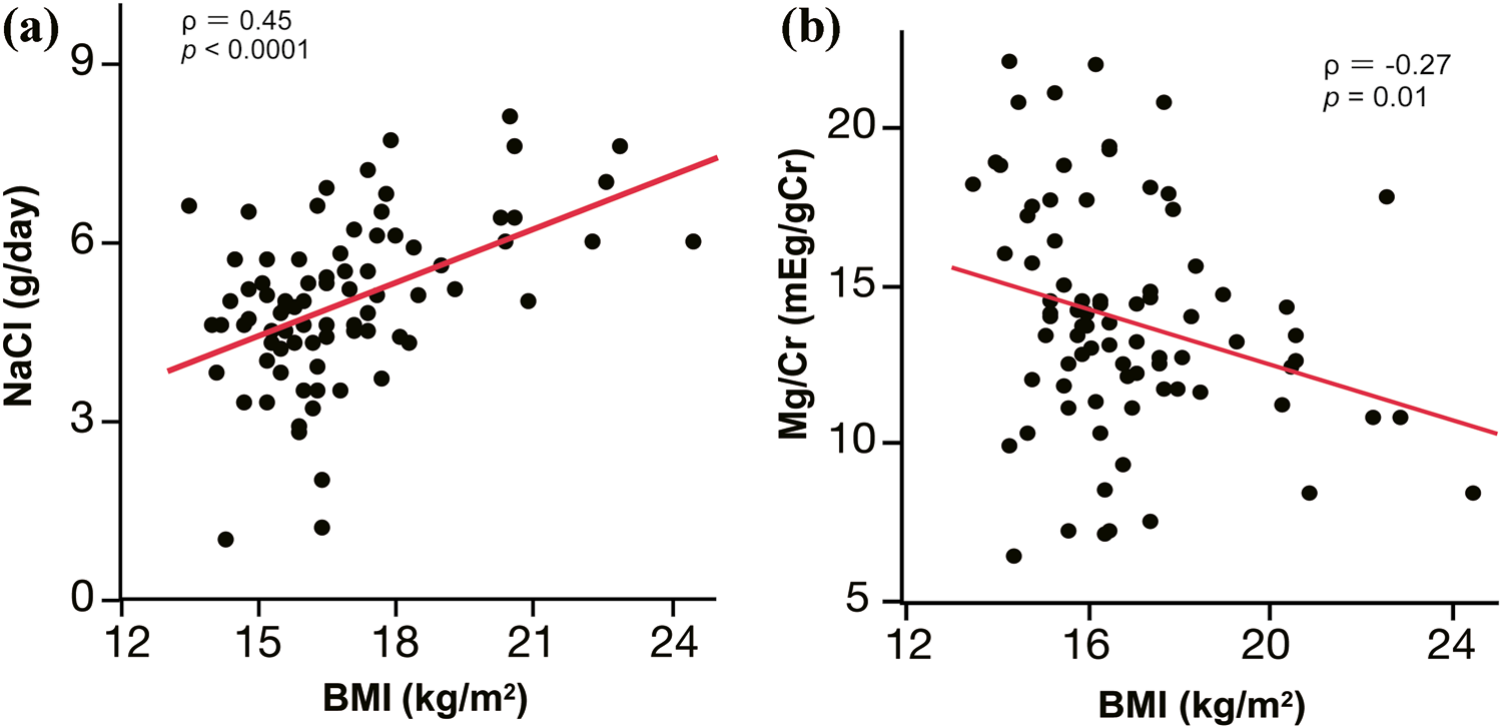
Spearman’s rank correlation coefficient showing significant correlations between BMI and estimated salt intake (**a**), and between BMI and urine Mg/Cr concentration (**b**), as evaluated by spot urine analyses

Table 3 shows anthropometric indices and blood pressure comparing healthy weight with obese/overweight children. Statistically significant differences were observed not only for weight, but also for height (healthy weight: 130.8 cm versus obese/overweight: 135.7 cm, *p* = 0.0053) (Table 3). However, blood pressure levels were apparently similar for healthy weight and obese/overweight children. NaCl intake was significantly different between healthy weight and obese/overweight children (healthy weight: 4.7 g/day versus obesity/overweight:6.0 g/day, *p* = 0.0001) (Table 4). As illustrated in Fig. 1, there was a positive correlation between BMI and NaCl (*ρ* = 0.45, *p* < 0.0001) (Fig. 1a) and a negative correlation between BMI and urinary Mg/Cr (*ρ* =  −0.27, *p* = 0.01) (Fig. 1b).

**Table 3:** Characteristics of obese/overweight and healthy-weight children

	Obese/overweight (*n* = 13)	Healthy-weight (*n* = 68)	*p* value
Height (cm)	135.7 ± 4.0	130.8 ± 5.8	<*0.01*
Weight (kg)	38.7 ± 4.9	27.6 ± 3.4	<*0.01*
BMI (kg/m^2^)	21.0 ± 1.7	16.1 ± 1.2	<*0.01*
BMI z-score^a^	1.9 ± 0.6	−0.2 ± 0.7	<*0.01*
Systolic BP (mmHg)	106 ± 9	104 ± 9	*0.41*
Diastolic BP (mmHg)	58 ± 6	57 ± 6	*0.68*

Values are shown as mean ± standard deviation (SD). Unpaired *t*-test was used to compare each characteristic in each weight group

^a^BMI values were converted to age-adjusted and sex-adjusted BMI z-score according to WHO 2007 references [18]

**Table 4:** Selected urine biomarkers and estimated salt (NaCl) intake in obese/overweight and healthy-weight children

	Obese/overweight (*n* = 13)	Healthy-weight (*n* = 68)	*p* value
pH	6.0 (5.625–6.0)	6.0 (5.875–6.25)	*0.01*
UN (mg/dL)	1301.6 ± 316.1	1222.1 ± 262.2	*0.33*
Cr (mg/dL)	122.6 ± 34.4	120.4 ± 38.7	*0.85*
Na/Cr (mEq/gCr)	108.7 (91.1–159.8)	119.6 (92.0–159.2)	*0.72*
K/Cr (mEq/gCr)	38.9 (23.5–43.1)	25.1 (21.4–34.1)	*0.07*
Mg/Cr (mEq/gCr)	12.4 (10.8–14.0)	13.9 (11.8–17.0)	*0.06*
NaCl (g/day)	6.0 (5.4–7.3)	4.7 (4.2–5.5)	<*0.01*
Na/K	4.2 (3.4–5.2)	4.7 (3.8–6.5)	*0.11*
	Obese/overweight (*n* = 13)	Healthy-weight (*n* = 62)	*p* value
Iso/Cr (nmol/mgCr)	13.9 (10.0–20.4)	20.1(10.5–35.4)	*0.21*
Tau/Cr (nmol/mgCr)	596.0 (482.5–956.9)	778.6 (579.3–1041.4)	*0.22*

Values are presented as median (25th and 75th percentiles) or mean ± standard deviation (SD). Wilcoxon test or unpaired *t*-test was used to compare each characteristic in each weight group

When performing a sensitivity analysis using only biomarker data from subjects’ urine samples that were collected across all four time points (*n* = 42), a moderate positive correlation between BMI and NaCl (*ρ* = 0.61, *p* < 0.0001) and slightly negative correlation between BMI and urinary Mg/Cr (*ρ* =  −0.23, *p* = 0.14) were found.

### Measurement of urinary biomarkers across time points

**Fig. 2:**
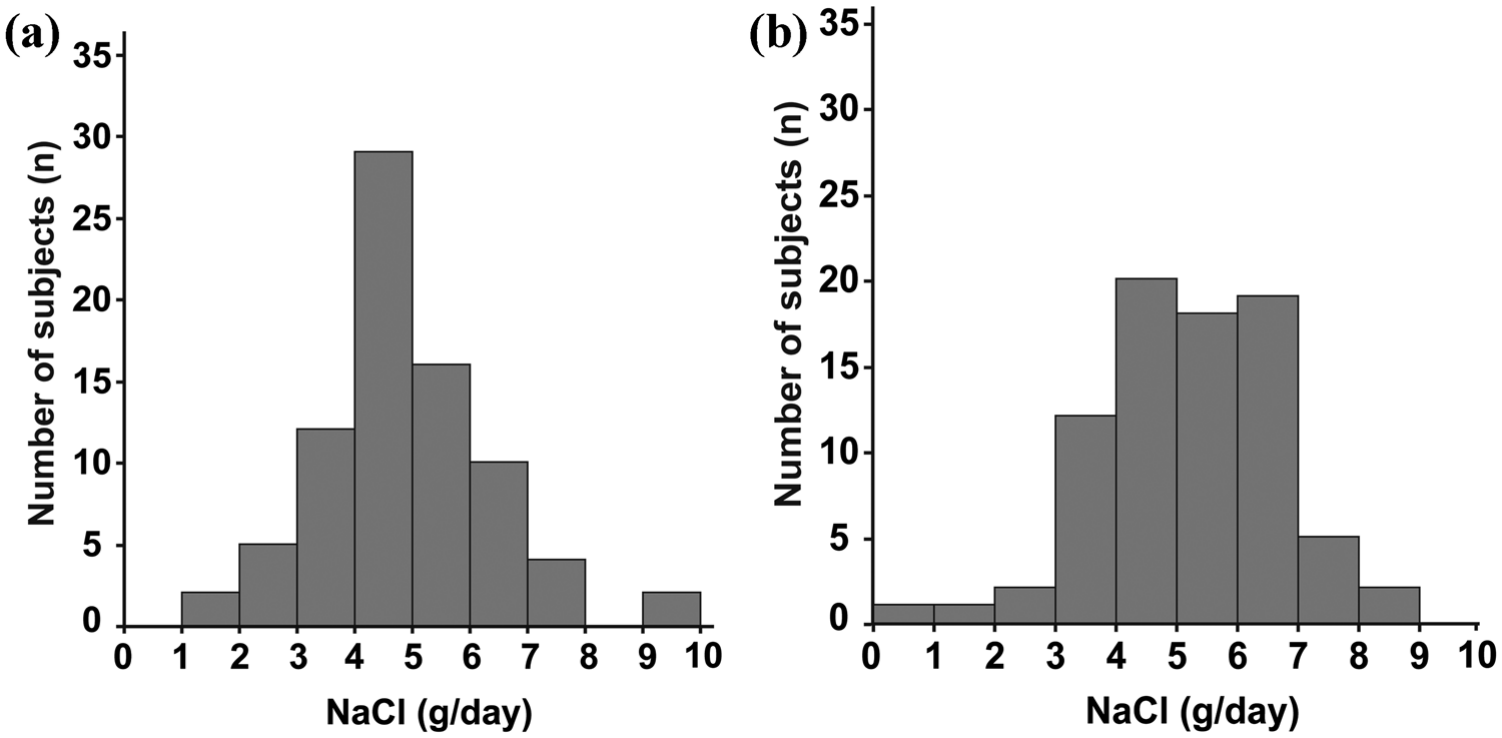
Distribution of the value of estimated salt intake on weekend (**a)** and on weekday (**b**), as evaluated by spot urine analyses and calculated using Tanaka et al.’s equation [17]

Both Na/Cr excretion level and Na/K ratio were significantly elevated in weekend as opposed to weekday samples (Na/Cr: 130.6 mEq/gCr versus 107.6 mEq/gCr, *p* < 0.0001), (Na/K: 5.4 versus 3.8, *p* < 0.0001), and Iso/Cr excretion level was significantly lower in weekend as compared to weekday samples (12.5 nmol/mgCr versus 21.5 nmol/mgCr, *p* = 0.0047) (Table 5). Although there were no statistically significant differences in estimated NaCl intake between weekend and weekday samples, the percentage of participants with NaCl intake estimated at ≥ 5 g/day was apparently greater in weekend as compared to weekday samples (weekend 54% versus weekday 38%) (Fig. 2).

**Table 5:** Selected urine biomarkers and estimated salt (NaCl) intake on weekend and weekday

	Weekend (*n* = 81)	Weekday (*n* = 81)	*p* value
pH	6.0 (6.0–6.5)	6.0 (5.75–6.25)	*0.09*
UN (mg/dL)	1250.8 ± 291.8	1218.9 ± 344.5	*0.53*
Cr (mg/dL)	123.1 ± 42.9	118.3 ± 43.1	*0.48*
Na/Cr (mEq/gCr)	130.6 (89.7–173.1)	107.6 (79.7–139.4)	*0.02*
K/Cr (mEq/gCr)	24.1 (17.3–34.7)	26.8 (221.3–38.4)	*0.06*
Mg/Cr (mEq/gCr)	14.9 (10.9–16.9)	13.4 (11.2–15.5)	*0.21*
NaCl (g/day)	5.2 (4.3–6.2)	4.8 (4.0–5.6)	*0.10*
Na/K	5.4 (3.9–7.3)	3.8 (2.9–5.0)	<*0.01*
	Weekend (*n* = 75)	Weekday (*n* = 75)	*p* value
Iso/Cr (nmol/mgCr)	12.5 (5.7–28.9)	21.5 (12.1–36.6)	<*0.01*
Tau/Cr (nmol/mgCr)	716.3 (422.6–1026.1)	781.0 (525.8–1028.4)	*0.32*

Values are presented as median (25th and 75th percentiles) or mean ± standard deviation (SD). Wilcoxon test or unpaired *t*-test was used to compare each characteristic in groups as classified according to sample collection time points

## Discussion

The present study showed a moderately significant positive correlation between NaCl intake and BMI, and a slightly significant negative correlation between Mg/Cr excretion and BMI in Okinawan schoolchildren. Research in pooled urine samples in humans has demonstrated that Na concentration reflects salt intake, K concentration reflects the intake of vegetables and fruits, and Mg concentration reflects the intake of unrefined whole grains such as brown rice, seeds, and seaweeds [19–21]. Although 24-h urine collection has been the standard that accurately captures the profile of dietary intake, in recent years, it has been reported that highly reproducible results can be obtained with spot urine collection and analytic methodologies [17, 22–24]. Based on these notions, in the present study of spot urine analyses, we did observe a number of significant differences in measurements of specific urinary biomarkers and estimated dietary micronutrient intakes in Okinawan schoolchildren when considering gender, BMI, and weekend versus weekday sampling. Regarding gender differences, boys showed a significantly higher value of urinary Na/Cr and K/Cr than girls. These results are in agreement with a larger sized study which recruited 766 Italian boys and 658 girls aged 6–18 years at National Health Service Centers across 10 regions, and 24-h urine collection found that boys across all developmental ages showed a significantly higher value of estimated NaCl and K intakes than girls [20]. As the children in our study were of pre-pubescent age, it is unlikely that such a gender dimorphism is due to sexual development or subsequent influence of sex hormones, but presumably more so a consequence of the increased amount of food boys consume as compared to girls [25].

When stratified subjects according to BMI in spot urine analyses, we observed significantly lower urine pH and significantly higher estimated NaCl intake in obese/overweight children compared to healthy weight children. In support of this, a cross-sectional study employing 5430 Japanese subjects using fasting single spot urine found that subjects with components of the Metabolic Syndrome were significantly associated with lower urine pH [26]. On top of this, 524 children (aged 6–17 years) in the Dortmund Nutritional and Anthropometric Longitudinally Designed (DONALD) Study in Germany also reported a significant inverse relationship with fat mass and urinary pH [27]. Concerning the elevated NaCl intake in obese/overweight children observed in our study, this is in line with a number of previous studies showing that NaCl intake is higher in obese and overweight adults as well as children [28–32].

Following on from this point, the level of estimated NaCl intake in spot urine analyses was positively associated with BMI in Okinawan schoolchildren. This finding is consistent with a number of previous larger, population-based studies in adults showing that NaCl excretion was positively associated with body weight as well as central obesity even after controlling for energy intake [33–35]. Other previous studies also observed the same results in children [36, 37]. Specifically, a large study with 1738 boys aged 10–18 years from the Korea National Health and Nutrition Examination found that subjects in the highest quartile for urinary sodium excretion to urinary gravity specific ratio (a representative for sodium intake) showed significantly higher body weight, BMI, and waist circumference among groups [37]. Also, a cross-sectional study of 374 Iranian children aged 11–18 years found that 24-h urinary Na/K ratio showed significantly positive associations with value of waist circumference and percentage of body fat even after adjusting for sugar sweetened beverage consumption or calorie intake [36]. Although our study cannot rule out the possibility that estimated higher NaCl intake in children with higher BMI is due to increased consumption of highly processed, energy dense foods, a line of plausible mechanisms has been proposed regarding the independent relationship between NaCl intake and overweight/obesity. Firstly, high NaCl intake is associated with increased urinary cortisol [38, 39] also in children [40]. Mechanism-oriented studies in mice show that high NaCl intake can stimulate aldose reductase–fructokinase pathway in both liver and hypothalamus, causing endogenous fructose production, subsequent leptin resistance, and hyperphagia, resulting in obesity [41]. Such a mechanism has been commonly observed in humans with healthy subjects consuming diets high NaCl, prone to developing type 2 diabetes and non-alcoholic fatty liver disease (NAFLD) [41].

Noticeably, we found that the Mg excretion levels in spot urine were inversely associated with BMI in Okinawan schoolchildren. These findings are also supported by previous population-based studies indicating that lower intake of Mg is associated with higher BMI [42–44]. Although there has not been enough data in children linking urinary excretion level of Mg with body weight, lower levels of serum Mg were shown to be significantly lower in both obese children and adolescents compared to healthy weight controls [45]. Several mechanisms are postulated as to how lower levels of Mg in urine samples may potentially relate with increased body weight. Firstly, Mg deficiency is tightly linked with an increase in a number of inflammatory markers including CRP and TNF-α in obese humans [46]. Mechanism-oriented studies in animals showed that Mg deficiency leads to an inflammatory response primarily via increasing intracellular calcium, which in turn causes priming of immune cells such as leukocytes and macrophages, resulting in a variety of inflammatory responses [47]. In addition, Mg is crucial for regulating several key enzymes involved in glucose metabolism [48]. In particular, Mg deficiency increases anaerobic metabolites such as lactate within adipocytes, leading to an increased triglyceride storage and insulin resistance in adipose tissue [49, 50].

Intriguingly in the present study, a significant increase in urinary sodium and Na/K ratio as well as a significant decrease in level of urinary isoflavones was observed in the weekend compared to the weekday in spot urine samples. In support of our data, a study of Japanese preschool children employing spot urine collection found that the Na excretion level as well as Na/K ratio was significantly higher on the weekend compared to the weekday [51]. Similarly, a cross-sectional study of Australian schoolchildren assessing electrolyte intake by 24-h urine discovered that NaCl intake was roughly 1.0 g/day higher on non-school days versus school days [52]. Regarding the analysis of other measured biomarkers in urine, weekend Iso excretion levels in our study were comparable to previous studies in Japanese preschool children reporting that mean Iso intakes range between 12 mg/d and 14 mg/d [53]. This notion raises a possibility that weekday Iso levels are significantly higher due to the inclusion of school lunch or “Kyushoku” featuring isoflavone-rich soy foods in Japan, thereby contributing to overall improved diet quality [54]. In contrast, our study in spot urine samples observed no statistically significant differences in taurine excretion levels, a validated biomarker of taurine-rich seafood and fish intake, between weekend and weekday samples. These findings may be a reflection of the substantial lack of seafood and fish featured in the diets of Okinawan schoolchildren both during the weekday including school lunches and also on weekends. In fact, a study assessing seafood intake as measured by urinary excretion of Tau in schoolchildren in mainland Japan showed that Tau/Cr level was higher compared to levels observed in our study [23].

There are a couple of limitations in the present study. Firstly, without collection of dietary intake data using validated assessment tools, we are unable to report on the intercorrelations among urinary biomarkers, dietary intake, and level of obesity. However, it should be noted that our results are well consistent with previous studies that employed urine analyses in combination with validated dietary intake assessment tools [43, 44, 54]. Secondly, as this study was cross-sectional, causal inference cannot be assumed regarding the temporal link between obesity and intake of specific micronutrients, and also, limited number of subjects reduced the power of the study. Finally, our study population was comprised of children residing in the southern island community of Japan with a distinctly different climate and culture compared to other Japanese communities and therefore should be taken this point into consideration.

## Conclusion

In Okinawan schoolchildren, high dietary intake of NaCl and low intake of Mg as estimated by spot urine analyses were significantly associated with body weight, suggesting a pivotal role of dietary electrolyte intake in the management of healthy body weight. Also, the lower dietary intake of NaCl on weekdays as opposed to weekends may suggest the positive influence of weekday school lunches in this population group. Overall, our results open up a potentially fresh avenue to expand food-based education programs in schoolchildren at the risk of obesity, further supporting the significance of Japanese school lunch in helping to improve the overall diet quality of children.

## Data Availability

The datasets generated during and/or analyzed during the current study are available from the corresponding authors on reasonable request.

## Abbreviations

BMI: Body mass index
BP: Blood pressure
UN: Urea nitrogen
Cr: Creatinine
CRP: C-reactive protein
HPLC: High-performance liquid chromatography
Iso: Isoflavones
K: Potassium
Mg: Magnesium
Na: Sodium
NaCl: Sodium chloride (salt)
SD: Standard deviation
Tau: Taurine
TNF-α: Tumor necrosis factor-alpha
WHO: World Health Organization
